# Alkali-Activated Red Mud and Construction and Demolition Waste-Based Components: Characterization and Environmental Assessment

**DOI:** 10.3390/ma15041617

**Published:** 2022-02-21

**Authors:** Alessio Occhicone, Mira Vukčević, Ivana Bosković, Serena Mingione, Claudio Ferone

**Affiliations:** 1 Department of Engineering, University of Naples ‘Parthenope’, Centro Direzionale, Isola C4, 80143 Napoli, Italy; claudio.ferone@uniparthenope.it; 2 Faculty of Metallurgy and Technology, University of Montenegro, Džordža Vašingtona bb, 81000 Podgorica, Montenegro; mirav@ucg.ac.me (M.V.); ivabo@ac.me (I.B.); 3 Laboratory for Concrete and Asphalt Chemistry, Empa Swiss Federal Laboratories for Materials Science and Technology, 8600 Dübendorf, Switzerland; serena.mingione@empa.ch

**Keywords:** red mud, blast-furnace slag, construction and demolition wastes, geopolymer-based materials, pavement blocks, LCA

## Abstract

The aluminum Bayer production process is the most diffused process in the world, but it creates a high amount of basic waste material known as red mud (RM). The use of RM as a precursor of alkali-activated materials is one of the best opportunities for both the ecosystem and the economy. In the present work, mortar samples were obtained by alkali activation of RM with various percentages of blast-furnace slag (BFS) and inert construction and demolition sands. This process creates samples that have a low environmental impact and that can be used as an alternative in the construction industry to cement materials or ceramic ones. The development of these new materials could also represent a way to reduce the CO_2_ emissions linked to cement and ceramic brick production. In the present study, cubic 40 mm samples reported very interesting values in compressive strength, with a maximum of about 70 MPa for low environmental impact mortars. With such a material, it is possible to create solid bricks for structural use and concrete tiles for road paving or use it for other purposes. Mortar specimens were prepared and characterized, and an LCA analysis with a “cradle-to-gate” approach was carried out for a comparison of the environmental impact of the studied mortars with other materials currently marketed.

## 1. Introduction

The production of building materials from bauxite residue, also called red mud (RM), has several potential benefits. RM is an unavoidable residue resulting from the first stage of aluminum Bayer production, and, as such, its management has long been an environmental problem for the global alumina industry [1,2].

Diverting RM from current landfill or lagoon practices and utilizing it as a secondary resource are important goals for the future of RM management, in consideration of the great quantity of this waste produced every year [3]. Recycling RM into building materials offers a high-volume valorization pathway for this material. At the same time, there is increasing demand from the construction industry for building materials with high environmental performance in a circular economy system [4]. The use of industrial residues [5,6], such as RM, as a replacement for virgin materials in the production of building materials has this logical approach. The European Commission has set out an EU-wide action plan for a circular economy at the government level (European Commission, 2015). This document describes the importance of reintegrating secondary raw materials into the economy, and it commits to promoting innovative industrial processes in which the wastes of one industry become the inputs to another [7].

In this study, we investigate the possibility of using a high volume of RM as a precursor in the building industry and, in particular, to produce inorganic alkali-activated components, such as bricks and blocks. Starting from wastes (in this case, secondary raw material), different precursors could be used to create cement-free jersey blocks, pavement blocks or bricks [8]. The use of RM and blast-furnace slag (BFS) can reduce the environmental impact of cement, which is mainly associated with the content of clinker, whose production is responsible on a global scale for about 10% of CO_2_ emissions [4]. It is estimated that only 3–4 million tonnes are used annually in cement production [9], in road construction [10] and as a source of iron compared to an annual production of 175 million tonnes [3].

In this framework, the use of alternative low-energy and low-CO_2_ binders, such as geopolymers and alkali-activated binders, is highly promising. Geopolymers have been proposed for a wide range of engineering applications, such as the production of geopolymer concrete [11]; the production of bricks and other non-structural elements starting from “poor” solid precursors [12]; hazardous waste stabilization/solidification [13,14]; fire-resistant binders [15,16]; and other functional applications [17,18,19].

Numerous recent studies investigated the use of RM as a precursor of alkali-activated materials [7,20,21,22]. The low percentage of silica and alumina in its composition makes RM unsuitable as the sole precursor, so other aluminosilicates must be added [23], but its intrinsic alkalinity promotes the activation. The produced materials have characteristics comparable to those of cement-based hydraulic binders; in fact, they show a good compressive strength and a good resistance to aggressive environments, and they can be produced at room or slightly higher temperature [24].

In this paper, a new mixture composition and a curing schedule for the use of bauxite residues as a co-precursor in the production of alkali-activated materials are presented. The binder was studied in previous work [25] by combining red mud (RM) with ground-granulated blast-furnace slag (BFS) activated by sodium silicate (SS) solution and different amounts of sodium hydroxide in alkaline solution.

In the preparation of the mortars, following the principle of the maximum reuse of materials, construction and demolition wastes were used as aggregate as an alternative to silica sand, reusing materials that, until now, have been mainly disposed of in landfills. For this reason, the performance of the mortars with this type of aggregate was evaluated and compared with those made up of silica sand. All the materials were studied for their environmental impact using a Life Cycle Assessment (LCA) with the cradle-to-gate approach.

In particular, the production of mortars with construction and demolition aggregates allows bases to be laid to bypass the critical points for a practical and extended commercial application of geopolymer-based products due to the use of a low-cost raw material and a low-energy-consumption process, which makes the final products competitive both from an environmental and economic point of view.

The main objective of this work was to develop eco-friendly components produced by a process that allows waste raw materials to be reused and valorized, following the circular economy philosophy. This environmentally friendly approach could contribute to creating, developing and optimizing technologies to close the loop for wastes (red mud and construction and demolition residues) and turning them into resources, with a full implementation of the circular economy policy, as also envisaged at the government level [26]. Construction and demolition waste materials are widely available, considering that they are generally stored in landfills or used as filler in the background for road surfaces [27]. In this work, cylindrical and cubic mortar samples were produced, analyzed and compared. The produced mortar blocks were suitably evaluated as alternatives to ceramic bricks, concrete and cement mortars for possible wide use in the construction industry.

## 2. Materials and Methods

Granulated blast-furnace slag (BFS) was supplied by ECOCEM, Fos-sur-Mer, France.

Sodium silicate (SS), R = 3.4 (SiO_2_:Na_2_O = 3.4, Na_2_O 7.5–8.5%, SiO_2_ 25.5–28.5% and ρ = 1.347 g/cm^3^), was supplied by Prochin Italia S.r.l. (Marcianise (CE), Italy) The R ratio was modified by adding anhydrous sodium hydroxide from Sigma Aldrich (Milano (MI), Italy), >98% purity pellets, 24 h before the preparation of samples.

Red mud (RM) from the Podgorica (Montenegro) plant was dried and ground with d_sauter_ = 10 µm.

Construction- and demolition-derived aggregates were provided by I.P.S. s.r.l. (San Martino Valle caudina (AV), Italy); in particular, washed sand (IPS-W) and raw sand (IPS-R) with a 0–4 mm particle size were used. The chemical composition is shown in Table 1.

Normalized silica sand was used as a comparison.

Recent studies demonstrated that Energy Dispersive X-ray Spectroscopy (EDS) large-area mapping can be successfully applied to obtain the chemical characterization of amorphous and crystalline phases [28]. According to the following procedure, the chemical composition of used materials was measured by EDS analysis: powder samples to be analyzed were ground to <100 μm size, homogenized and pressed to form a disk with a ≈1 cm diameter. The obtained disk was examined for each sample in five different areas, each with a surface of about 1 mm^2^. The results reported in Table 1 are the average of five measurements of EDS analysis performed on the selected area for all analyzed powder fractions [25,28].

In order to determine the basicity of the RM sample, a pH analysis in an aqueous solution was carried out using a Hanna instrument pH 211 pH meter (METTLER TOLEDO, Torino (TO), Italy), calibrated at three pH points of 4, 7 and 10. Measurements were made at regular intervals of 5 min in a range of 0–25 min for samples with a solid/liquid 1:30 ratio. The result obtained was 10.55 ± 0.05.

Wide-angle X-ray diffraction patterns were obtained at room temperature with an automatic Rigaku powder diffractometer Miniflex 600 (Rigaku, Tokyo, Japan) operating in the θ/2θ Bragg–Brentano geometry and using CuKα radiation (1.54 Å,) 40 kV, 15 mA, 2θ range from 5° to 80°, step size 0.020° 2θ and 0.5 slit width. The phase recognition was carried out using the PDF-4+ 2021 database (International Centre for Diffraction Data^®^, Newtown Square, PA, USA) and Rigaku PDXL2 software (Rigaku, Tokyo, Japan).

Particle size distribution data values were obtained with the Mastersizer 3000 laser diffraction particle size analyzer (Malvern Panalytical, Monza (MB), Italy).

Scanning electron microscopy (SEM) microstructural analysis was performed by using a Phenom Pro X microscope (Thermo Fisher Scientific, Rodano (MI), Italy) on freshly prepared fracture surfaces. The same instrument was used for energy-dispersive X-ray spectroscopy (EDS) analysis.

The FTIR absorption spectra were recorded in the range of 4000–400 cm^−1^ using a Nicolet system, model Nexus, equipped with a DTGS KBr detector (deuterated triglycine sulfate with potassium bromide windows). A spectral resolution of 2 cm^−1^ was chosen.

Mechanical sample analysis was performed with Controls MCC8 hydraulic console (Controls, Milano (MI), Italy) with 2000 kN capacity according to UNI EN-196-1: 2016.

### 2.1. Preparation of the Samples

Starting from previous experience with RM/BFS activation [25], the procedure for sample preparation involved the following steps:

- Addition of RM powder to sodium silicate solution;
- Addition of BFS in a small amount to the mixture with a high level of stirring;
- Addition of aggregate in saturated surface dry (SSD) condition.

Mortar samples were cast in cube molds, with 40 mm edges, or cylindrical molds, d = 27 mm and h = 50 mm. Two different curing conditions were tested: (i) at room temperature with 95% humidity for 7 d and after 21 d at room temperature condition; (ii) at 40 or 60 °C with 95% humidity in the oven for 24 h, 6 d at room temperature with 95% humidity and after 21 d at room temperature and humidity conditions. The composition and the label of the different mixtures tested are reported in Table 2.

### 2.2. Life Cycle Assessment

One of the goals of the study was to perform a comparative Life Cycle Analysis on RM/BFS-C&D sand (M70_W_RT), RM/BFS silica sand (M70_sand_RT) mortars and cement-based analogues (OPC). The LCA was performed according to ISO14040:2006 corresponding to the requirements of the guidelines concerning Type III declarations [29].

In this study, the system boundary involves raw materials, transport, manufacturing and disposal (available for use). In detail, the LCA was assessed to consider the contribution of the raw materials to all the systems, as far as the production processes. The contribution of the energy consumption of the steps in common, as occurs for material mixing, was considered the same for all the processes (no difference in workability was estimated); the energy consumption for the milling step was only considered for RM mechanical activation; the contribution of energy for clinker and BFS milling are already evaluated in material production.

**Figure 1:**
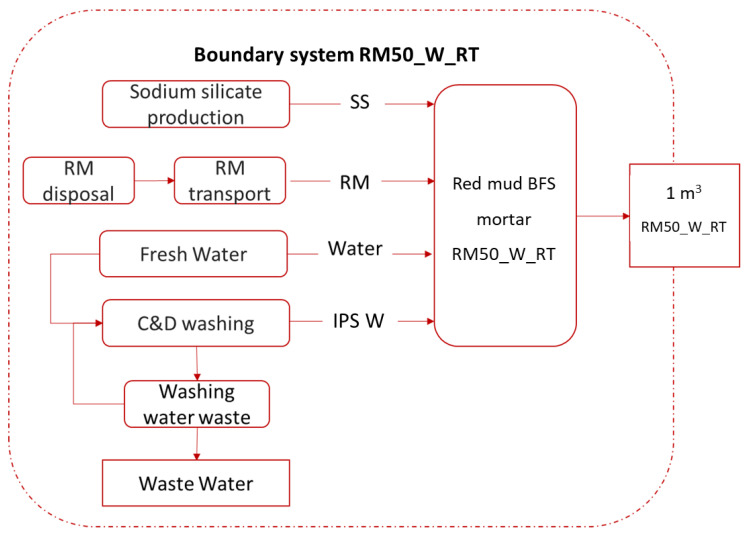
RM_W_RT sample boundary system for LCA analysis cradle-to-gate approach.

It must be noted that raw materials (A1), transport (A2) and manufacturing processes (A3) are included in the LCA calculation according to PCR rule EN 15804:2013, clause 6.3.4 [18] (example of boundary system in Figure 1).

The transport stage was considered for the delivery of the raw materials to the plant, considering an average transfer of 100 km by Euro 5 heavy vehicles for the estimation in Italy [18].

It is worth noting that this estimation had little effect on the overall production impacts of each system, bordering on undetectable [19]. To be sure that the assumption made was verified, a simulation of the environmental impacts resulting from the shift of the red mud from the Podgorica site to the sand production site was also carried out, but the final environmental impact value increased by less than 5%, justifying the use of uniform transfer values for all materials used.

No treatment of “the end of life” was considered for all the products, using a “cradle-to-gate” approach. The use phase and disposal phase of the product are omitted in this case.

According to the LCA approach, the *functional unit* was defined to provide a reference to which the inputs and outputs could be related [30]. The functional unit is 1 m^3^ of mortar produced in relation to the respective calculated apparent density (Table 3).

#### 2.2.1. Inventory Analysis and Criteria

This step of the LCA involves the collection of data for each unit process regarding all relevant inputs and outputs of energy and mass flow, as well as data on emissions to air, water and land. All data about the material and energy flows considered in the study were obtained from various sources, e.g., from available datasets (included in the SimaPro© software v.8.52, 2b, Mogliano Veneto (TV), Italy) and from the literature data. Missing, incomplete or non-accessible data were completed by secondary data, such as Ecoinvent v.3 databases, literature reviews and expert judgements [18].

All operating data, namely, the starting material, thermal energy, internal fuel consumption and electricity consumption, waste production and all emission measurements available, were taken into consideration in the analysis. If no primary data were available, assumptions were made as shown for the transport distances. At the same time, all material and energy flows with a share of less than 1% were also considered. It can be assumed that the total of all neglected processes does not exceed 5% in the effective categories. In this evaluation, the spaces, machinery, plants and infrastructure required for the realization of the manufacturing process were ignored.

#### 2.2.2. ReCiPe 2016 Endpoint Method

The environmental impact category indicators calculated in this paper were referred to as endpoint level based on ReCiPe 2016 endpoint Hierarchist version, with European normalization and average weighting set [31].

**Figure 2:**
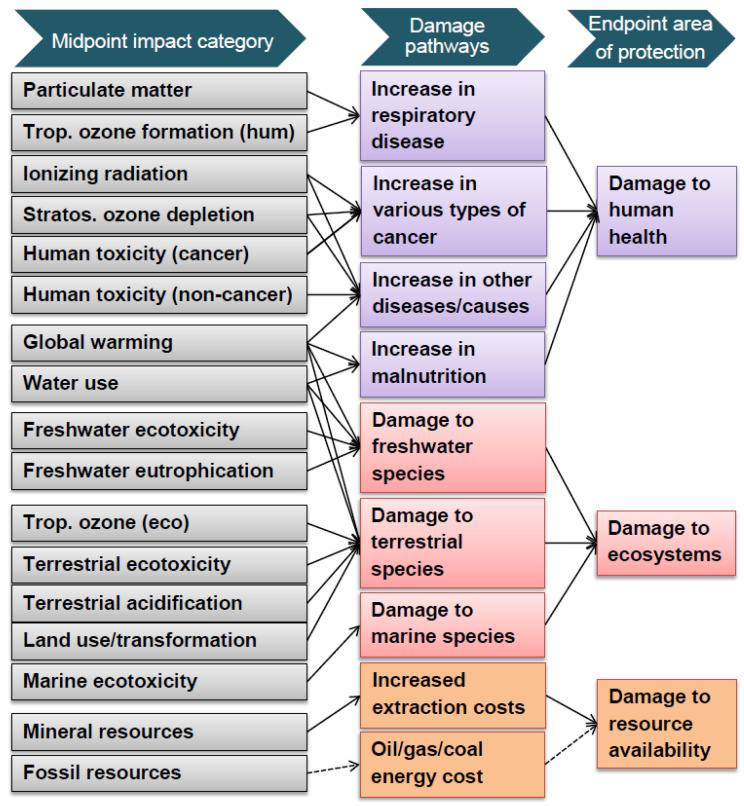
ReCiPe LCA method at different categories of impact levels [31]. Reprinted with permission from Ref. [32]. Copyright ReCiPe 2016 National Institute for Public Health and the Environment.

Endpoint indicators (or damage approach) show the environmental impact on three higher aggregation levels starting from 17 impact categories, namely, the (1) effect on human health, (2) biodiversity and (3) resource scarcity (summarized in Figure 2).

This type of methodology was adopted, which, starting from the impact categories used for the elaboration of an environmental product declaration (EPD) (an independently verified and registered document that communicates transparent and comparable information about the life cycle environmental impact of products in a credible way [33]), provides results in one score, resulting from the addition of the three categories shown in Figure 2. Environmental impacts and the economic expenditures necessary for the production of the materials were assessed according to the compositions shown in Table 3.

**Table 3:** RM/BFS mortars and standardized mortar chemical composition for LCA analysis. All the amounts are related to 1 m^3^ of material production.

	Alkali-Activated Material			OPC Material
Materials (kg)	M50_W_RT	M50_sand_RT	M75_Wnew_RT	OPC Mortar [34]
Red mud	287	294	430	-
Blast-furnace slag	287	294	141	-
Sodium silicate	258	265	151	-
Sand	-	1176	-	1370
IPS sand	1149	-	1145	-
Cement (Portland)	-	-	-	420
Water	68	71	208	300
Density (kg/m^3^)	2050	2100	2075	2090

In addition to the materials shown in Table 3 with the relative compositions and density, the materials present in the Ecoinvent 3.0 database that were already present in SimaPro version 8.52 were analyzed for comparison, namely, sand–lime bricks, concrete blocks and solid ceramic bricks; 1 m^3^ environmental impact production were tested.

## 3. Results

### 3.1. Compressive Strength

The first series of experiments was conducted on cylinders with a 27 mm base and 50 mm height (height/diameter ratio close to 2). These samples were made to evaluate the dependence of the mechanical strength of the mortars on the curing conditions. Tests were carried out with the standard silica sand and the two different sand samples resulting from construction and demolition waste, with the same percentage of RM and BFS in the powder mix (50:50).

**Figure 3:**
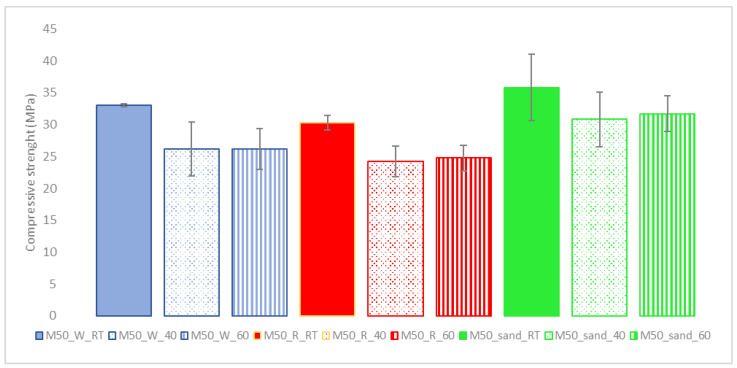
Compressive strength values of mortar cylindrical specimens with an RM/BFS ratio of 50/50 and three different sands (see Table 2) at different curing temperatures.

In Figure 3, it is possible to observe that the three mortars show a similar dependence on the curing temperature. In particular, the materials treated at room temperature show average values of strength, which are higher than those of the homologs treated at higher temperatures (the values of the mortars treated at 40 and 60 °C). The trends in compressive strength relative to the curing temperature are quite similar to those previously studied [25]. Even in this case, by increasing the curing temperature, the reaction kinetics (especially those involving reactive calcium compounds contained in blast-furnace slag) increased, limiting the contribution to the material’s strength due to the formation reactions of the CSAH phases and Si–O–Al bonding resulting from the activation of the red mud [20,25].

No large deviations are observed between the three types of sand; the mortars with silica sand show compressive strength values about 2 MPa higher than those of washed IPS sand and about 5 MPa higher than those of IPS raw sand.

**Figure 4:**
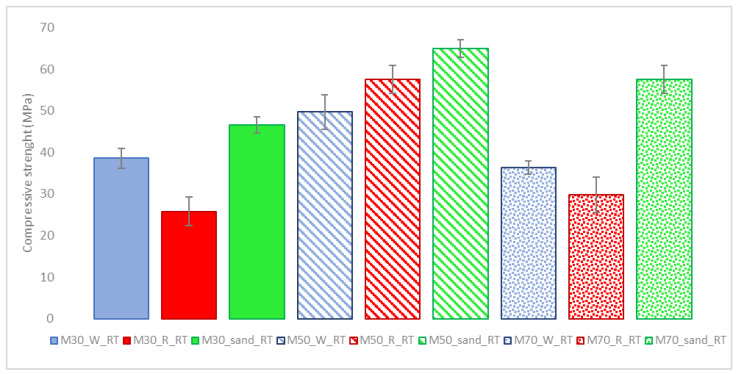
Compressive strength values of mortar specimens with different amounts of RM in the powder mixture. Average compressive strength resistance on 3 cubic samples of mortars cured at room temperature.

New mixtures with different RM and BFS contents were made to assess how an increase in the quantity of RM could influence the performance of the produced mortars at room temperature. In order to obtain strength values to be easily compared with those from the literature data, cubic specimens, with 40 mm sides, were prepared in this phase. Figure 4 shows the average values of three tests for each cubic sample cured at room temperature, using three RM/BFS ratios (30:70, 50:50 and 70:30). The mortar samples produced with 100% RM showed no acceptable compressive strength values and are not reported in this paper.

The compressive strength values of the mortars follow the trends obtained for the pastes in the previous work. The anomalous behavior of the M50_R_RT sample may be due to the unavoidable heterogeneity of unwashed IPS raw sand. The obtained results are interesting because of the widespread use of these materials in the construction industry. In fact, the mortar with the lowest compressive strength recorded is the sample with a 30–70 RM/BFS ratio with recycled IPS raw sand (M30_R_RT), which recorded values close to 30 MPa. This result may seem surprising, since by adding a higher amount of RM, reducing the amount of slag, the strength values of the materials increase, also sensitively considering mixtures with 50% of RM (M50 species). These results confirmed a synergistic effect of RM/BFS in a 1:1 ratio, detected on pastes [25], with compressive strength values not sensibly decreasing when the amount of BFS decreases, provided it is present at least in a 3:1 ratio with RM.

The M70 series shows compressive strength values comparable to those of the M30 series, but slightly lower than those of the M50 series. In this case, the reactive component (BFS) responsible for the formation of the main structural network in the samples is present in a smaller quantity than the others. This implies that the amount of RM introduced into the mixture does not significantly reduce performance compared to a higher amount of BFS, and this is certainly encouraging for large-scale material use.

In all cases with the same RM/BFS ratio, the use of silica sand ensures better performance for the material. This is not surprising considering the unavoidable porosity of recycled sand and the possible interactions of calcium oxide present in recycled sand with the reagent matrix, limiting the synergistic effects seen for RM/BFS pastes [20,25].

To further reduce the environmental impact of mortar production, two new mixtures were added, in which part of the sodium silicate (about 40%) was replaced with water. For these types of mixtures, the total quantity of liquids was lower due to the lower viscosity of water, causing a workability increase in the new mixtures. The characterization of the two new mixtures, both prepared with washed IPS sand and two different RM/BFS ratios (50:50 and 75:25), provided interesting compressive strength values (57.5 MPa for the sample with a ratio of 50:50 and 42.6 MPa for the RM/BFS ratio of 75:25).

### 3.2. XRD

In addition to the mechanical characterization, a comparison was made of the phases formed and lost as a result of alkaline activation. A comparison was also made concerning the pastes processed in a previous work [25] to evaluate the possible crystalline phases derived from the construction and demolition sands used as aggregate.

**Figure 5:**
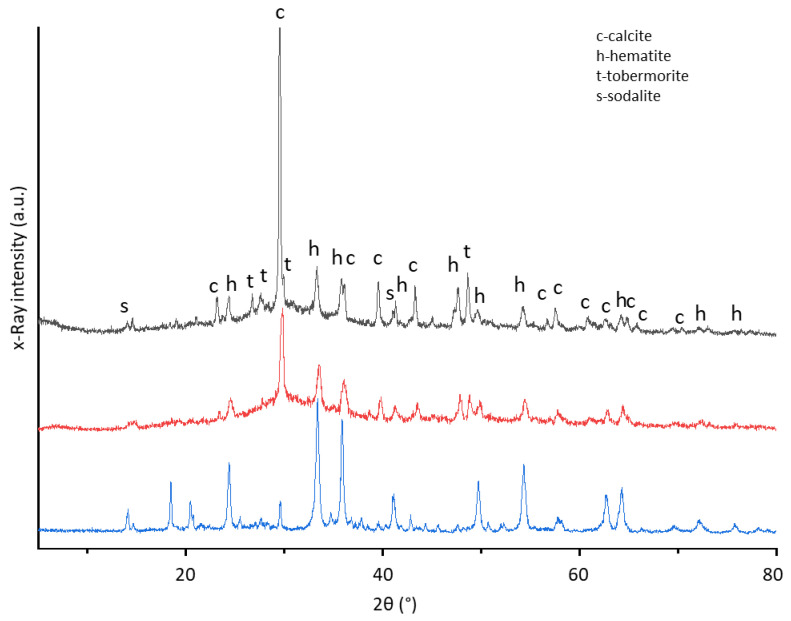
M50_W_RT XRD pattern with relative crystalline phases: c—calcite, h—hematite, s—sodalite and t—tobermorite (gray line) (the upper curve). Paste RMBFS50_RT (red line) (in the middle) [25] and RM (blue line) (the downward curve).

Figure 5 shows the diffraction patterns of RM as it is, the paste made with RM/BFS (50 RM and 50 BFS) cured at room temperature [25] and the M50_W_RT mortar with relative crystal phases. It is possible to see how some RM components, such as sodalite, boehmite and gibbsite, react, likely forming an amorphous phase with the SS solution, which can be observed both in the paste and in the mortar. Furthermore, the calcite peak appears around 2θ = 30° and has a greater intensity for the mortar than for the paste due to the high amount in washed IPS sand (as it also appears in the EDS analysis composition in the previous paragraph). It is also worth noting the appearance of the tobermorite peaks, related to the CSH phase. Under these peaks, a broad hump appears, which is likely attributable to the amorphous CSH/CSAH phase (also visible in the paste pattern). This is the phase that mainly contributes to the hardening of the material and, therefore, to its mechanical properties.

### 3.3. LCA Environmental Analysis of RM/BFS Mortars Compared with Other Brick and Block Materials

**Figure 6:**
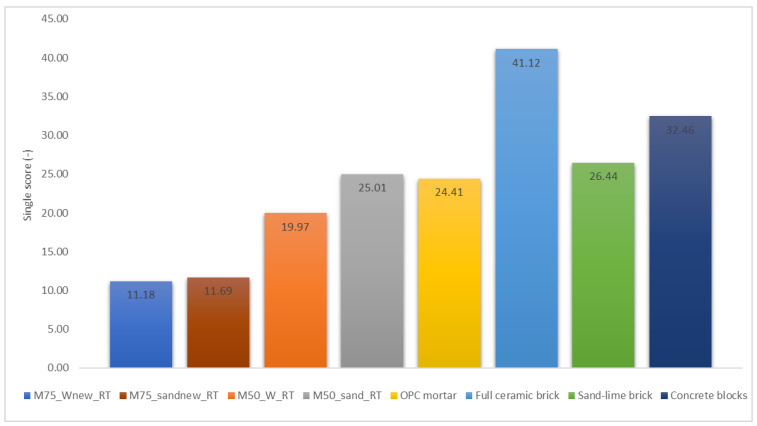
LCA was performed with the ReCiPe endpoint method of different materials relative to brick and block production. All impacts are relative to the single score of this method.

The ReCiPe hierarchic endpoint method allowed us to estimate the impact factors linked to each material production process with the relative contribution of transport and energy demand in the process up to the gate. With this method, all the categories’ contributions are summarized in a unique value (Figure 6).

The recorded values are very different depending on the species examined. The lower impact values are obviously preferrable and, looking at Figure 6, it is clear that the alkali-activated mortar called M75_Wnew_RT is significantly the least impactful material of the lot, together with its counterpart produced with silica sand. These samples were created precisely to minimize the environmental impacts of production. The material produced with recycled sand showed an average value of 42 MPa of compressive strength, while that produced with silica sand was slightly higher than 46 MPa. These values allow us to say that they can be safely used as eco-sustainable substitutes for the current bricks commonly used in the construction industry. In particular, the product M75_Wnew_RT foresees a wide use of RM with construction and demolition sands, which are thereby subtracted from the classic logic of waste disposal in landfills. However, encouraging values were also obtained for mortars with an RM/BFS 50:50 ratio, although the results in this case are very similar to those of standardized OPC mortar, taken as a reference for the obtained environmental impact values and sand–lime brick. In this case, the difference in environmental impact between the mortar produced with recycled sand and that produced with silica sand is greater because the latter also has a higher density, and, therefore, the production of 1 m^3^ of material is more impactful (all the impacts values are shown in Appendix A).

All the other materials, with which our products were compared, are significantly more impactful than RM/BFS mortars. This means that, depending on the desired performance features, the studied materials can be excellent eco-sustainable substitutes for current building materials. Among others, ceramic bricks are by far the worst for the consumption of environmental resources and for the consequent impact on greenhouse gases and marine ecosystems. This is mainly due to the high energy consumption required in the firing phase of the clayey material at temperatures above 1000 °C.

The concrete blocks also show a rather high overall value, mainly due to the high temperatures of clinker production and the CO_2_ released into the atmosphere by the calcination of clinker raw materials [35]. The overall impact is lower than that of ceramics due to a large amount of aggregate present in the mixture, which has a low impact on the environment (as is the case for sand in standard mortar).

The new alkali-activated products proposed in this work can therefore be used as prefabricated products, bricks and highway separation jersey, and they can be evaluated with the proper modifications as thermal/acoustic insulation materials, which are very topical nowadays in Italy [36] (D.M. 6 August 2020, implementation of Law 77/2020). The manufacturing plant of new components should be located in areas adjacent to steel and/or aluminum factories, because, in this way, raw materials can be easily sourced and transport impacts reduced. This is surely one of the limits to the diffusion of the proposed materials, especially in Italy, where there are no large industrial sites of this type. One of the few areas of interest could be the area between Puglia and Basilicata or Sardinia (rich in both BFS and RM). It should also be noted that bauxite residues (RM) are very different from each other; therefore, before starting a plant, it would be necessary to verify the composition of this precursor to reproduce mixtures with the appropriate mechanical, physical and chemical properties.

## 4. Conclusions

In this work, RM and BFS were tested as precursors for alkali-activated mortars in combination with an SS solution and different kinds of sand (construction and demolition wastes or silica sands). Each mixture was prepared with different percentages of precursors and different curing temperatures. The results confirm the effective applicability of bauxite residues from the Podgorica industrial site as a precursor to alkali-activated materials.

Synergistic behavior was found between RM and BFS, with compressive strength results higher than 60 MPa for samples with a 1:1 ratio between the two solid precursors.

Mortar samples produced with recycled aggregates showed high compressive strength, even with low percentages of blast-furnace slag (36 MPa for RM/BFS 70:30%wt); their strength values were slightly lower than those of mortars with silica sand.

The obtained results are quite important, especially considering that they have been obtained, in the best case, with a material containing only 7% of pure raw material (sodium silicate) plus 5.5% of tap water. Cubic samples were produced, showing more than 40 MPa compressive strength with a system composed of, if we also consider BFS as a “second raw material”, 80%wt of species nowadays destined to landfill or, at most, used as filler.

This is reflected in the environmental impact of the samples, which is half compared to the traditional alkali-activated materials or cement mortars that show similar behavior.

The tested mixture can be easily replicated using red mud of different origins, and it can be used in many ways by the construction industry with a much lower environmental impact than that of its traditional or commercial counterparts, together with a particular aesthetic that could be exploited by designers.

## Figures and Tables

**Table 1:** Chemical composition of RM, BFS, IPS-W washed C&D sand and IPS-R raw C&D sand expressed as the weight percentage of oxides from EDS analysis (IPS sands have about 18–19% of carbonate, which is not reported in the table).

Material	SiO_2_	Al_2_O_3_	Na_2_O	Fe_2_O_3_	TiO_2_	MgO	CaO	K_2_O
**RM**	15.5 ± 0.1	24.0 ± 0.4	8.1 ± 0.1	45.0 ± 0.2	5.4 ± 0.1	0.9 ± 0.1	0.8 ± 0.1	0.30 ± 0.05
**BFS**	35.7 ± 0.7	11.2 ± 0.1	-	0.3 ± 0.1	0.5 ± 0.1	6.5 ± 0.1	43.9 ± 0.8	0.31 ± 0.07
**IPS-W**	26.6 ± 0.3	9.2 ± 0.2	1.1 ± 0.1	-	-	-	44.4 ± 0.5	-
**IPS-R**	28.2 ± 0.6	12.2 ± 0.5	0.8 ± 0.1	-	-	-	41.6 ± 1.3	-

**Table 2:** RM/BFS mortar chemical composition (the first number refers to %wt. of RM in the RM/BFS precursor mixture). Reported in the table are the percentages of RM in powder mix; sodium silicate R-value is equal to 2. The Sand ratio is the ratio between the amount of inert material (sand) and the sum of the reagent powders (RM, BFS).

Name	Red Mud	Blast-Furnace Slag	Sand Ratio	Solid/Liquid	Curing Temperature
**M30_W_RT**	30	70	2	3	25
**M50_W_RT**	50	50	2	3	25
**M50_W_40**	50	50	2	3	40
**M50_W_60**	50	50	2	3	60
**M70_W_RT**	70	30	2	3	25
**M30_R_RT**	30	70	2	3	25
**M50_R_RT**	50	50	2	3	25
**M50_R_40**	50	50	2	3	40
**M50_R_60**	50	50	2	3	60
**M70_R_RT**	70	30	2	3	25
**M30_sand_RT**	30	70	2	3	25
**M50_sand_RT**	50	50	2	3	25
**M50_sand_40**	50	50	2	3	40
**M50_sand_60**	50	50	2	3	60
**M70_sand_RT**	70	30	2	3	25
**M50_Wnew_RT**	50	50	2	2.9	25
**M75_Wnew_RT**	75	25	2	2.9	25

## Data Availability

Not applicable.

## Appendix A

**Table A1:** Values of the production process environmental impact for all materials compared in the work.

	Unit	M75_WNEW_RT	M75_SANDNEW_RT	M50_W_RT	M50_SAND_RT	OPC Mortar	Full Ceramic Brick	Sand–Lime Brick	Concrete Blocks
**Total**	Score	11.18	11.69	19.97	25.01	24.41	41.12	26.44	32.46
**Human Health**	Score	4.24	3.40	7.72	10.11	12.19	17.39	10.99	14.41
**Ecosystems**	Score	2.84	3.43	5.28	6.11	7.65	10.79	6.95	8.51
**Resources**	Score	4.09	4.86	6.97	8.79	4.57	12.93	8.50	9.55
**Density**	kg/m^3^	2075	2100	2050	2100	2090	1700	1850	2000

Life cycle assessment values of all the materials studied in the paper, with the relative contribution for all the three macro-categories of the ReCiPe 2016 endpoint method.

## References

[1] Evans K. (2016). The history, challenges, and New developments in the management and use of bauxite residue. J. Sustain. Metall..

[2] George E.T., MacKenzie S.D. (2003). Handbook of Alluminium, Physical Metallurgy and Process.

[3] (2018). World Aluminium. http://www.world-aluminium.org/statistics/alumina-production/.

[4] Monteiro P., Miller S., Horvath A. (2017). Towards sustainable concrete. Nat. Mater..

[5] Juenger M.C.G., Winnefeld F., Provis J.L., Ideker J.H. (2011). Advances in alternative cementitious binders. Cem. Concr. Res..

[6] Lothenbach B., Scrivener K., Hooton R.D. (2011). Supplementary cementitious materials. Cem. Concr. Res..

[7] Joyce P.J., Hertel T., Goronovski A., Tkaczyk A.H., Pontikes Y., Björklund A. (2018). Identifying hotspots of environmental impact in the development of novel inorganic polymer paving blocks from bauxite residue. Resour. Conserv. Recycl..

[8] Messina F., Ferone C., Molino A., Roviello G., Colangelo F., Molino B., Cioffi R. (2017). Synergistic recycling of calcined clayey sediments and water potabilization sludge as geopolymer precursors: Upscaling from binders to precast paving cement-free bricks. Constr. Build. Mater..

[9] Pontikes Y., Angelopoulos G.N. (2013). Bauxite residue in Cement and cementious materials, Resources. Conserv. Recycl..

[10] Biswas W.K., Cooling D.J. (2013). Sustainability Assessment of Red Sand as a substitute for Virgin Sand and Crushed Limestone. J. Ind. Ecol..

[11] Nath P., Sarker P.K. (2014). Effect of GGBFS on setting, workability and early strength properties of fly ash geopolymer concrete cured in ambient condition. Constr. Build. Mater..

[12] Messina F., Ferone C., Colangelo F., Cioffi R. (2015). Low temperature alkaline activation of weathered fly ash: Influence of mineral admixtures on early age performance. Constr. Build. Mater..

[13] Ferone C., Colangelo F., Messina F., Santoro L., Cioffi R. (2013). Recycling of pre-washed municipal solid waste incinerator fly ash in the manufacturing of low temperature setting geopolymer materials. Materials.

[14] Mohammadinia A., Arulrajah A., Sanjayan J., Disfani M.M., Win Bo M., Darmawan S. (2016). Stabilization of demolition materials for pavement base/subbase applications using fly ash and slag geopolymers: Laboratory investigation. J. Mater. Civ. Eng..

[15] Colangelo F., Roviello G., Ricciotti L., Ferone C., Cioffi R. (2013). Preparation and characterization of new geopolymer-epoxy resin hybrid mortars. Materials.

[16] Roviello G., Ricciotti L., Ferone C., Colangelo F., Tarallo O. (2015). Fire resistant melamine based organic-geopolymer hybrid composites. Cem. Concr. Compos..

[17] Colangelo F., Roviello G., Ricciotti L., Ferrandiz-Mas V., Messina F., Ferone C., Tarallo O., Cioffi R., Cheeseman C.R. (2018). Mechanical and thermal properties of lightweight geopolymer composites. Cem. Concr. Compos..

[18] Ricciotti L., Occhicone A., Petrillo A., Ferone C., Cioffi R., Roviello G. (2020). Geopolymer-based hybrid foams: Lightweight materials from a sustainable production process. J. Clean. Prod..

[19] Frattini D., Occhicone A., Ferone C., Cioffi R. (2021). Fibre-Reinforced Geopolymer Concretes for Sensible Heat Thermal Energy Storage: Simulations and Environmental Impact. Materials.

[20] Lemougna P.N., Wang K., Tang Q., Cui X. (2017). Study on the development of inorganic polymers from red mud and slag system: Application in mortar and lightweight materials. Constr. Build. Mater..

[21] Boskovic I.V., Nenadovic S.S., Kljajevic L.M., Vukanac I.S., Stankovic N.G., Lukovic J.M., Vukcevic M.A. (2018). Radiological and physicochemical properties of red mud based geopolymers. Nucl. Technol. Radiat. Prot..

[22] Tsakiridis P.E., Agatzini-Leonardou S., Oustadakis P. (2004). Red mud addition in the raw meal for the production of Portland cement clinker. J. Hazard. Mater..

[23] Luukkonena T., Abdollahnejada Z., Yliniemia J., Kinnunena P., Illikainena M. (2018). One-part alkali-activated materials: A review. Cem. Concr. Res..

[24] Nevill A.M. (2011). Properties of Concrete.

[25] Occhicone A., Vukcevic M., Boskovic I., Ferone C. (2021). Red Mud-Blast Furnace Slag-Based Alkali-Activated Materials. Sustainability.

[26] European Commission (2020). A New Circular Economy Action Plan.

[27] Petrillo A., Cioffi R., Ferone C., Colangelo F., Borrelli C. (2016). Eco-sustainable Geopolymer concrete blocks production process. Agric. Agric. Sci. Procedia.

[28] Alam Q., Schollbach K., van Hoek C., van der Laan S., de Wolf T., Brouwers H.J.H. (2019). In-depth mineralogical quantification of MSWI bottom ash phases and their association with potentially toxic elements. Waste Manag..

[29] ISO 14025:2006 Environmental Labels and Declarations—Type III Environmental Declarations—Principles and Procedures. https://www.iso.org/standard/38131.html.

[30] Nwodo M.N., Anumba C.J. (2019). A review of life cycle assessment of buildings using a systematic approach. Build. Environ..

[31] LCIA: The ReCiPe Model, RIVM Committed to Health and Sustainability. 11 February 2018. https://www.rivm.nl/en/life-cycle-assessment-lca/recipe.

[32] Huijbregts M.A.J., Steinmann Z.J.N., Elshout P.M.F., Stam G., Verones F., Vieira M., Zijp M., Hollander A., van Zelm R. (2017). ReCiPe2016: A harmonised life cycle impact assessment method at midpoint and endpoint level. Int. J. Life Cycle Assess..

[33] Environdec, Type III Environmental Declaration (ISO 14025), The International EPD System. https://www.environdec.com/home.

[34] Pacenti V. (1993). The Mortars.

[35] Telschow S., Frandsen F., Theisen J., Dam-Johansen K. (2012). Cement formation-A success story in a black box: High temperature phase formation of Portland cement clinker. Ind. Eng. Chem. Res..

[36] D.M. MISE 6 August 2020 Implementation of Law 77/2020. Italy. https://www.gazzettaufficiale.it/eli/id/2020/10/05/20A05394/sg.

